# The National Eclipse Weather Experiment: an assessment of citizen scientist weather observations

**DOI:** 10.1098/rsta.2015.0220

**Published:** 2016-09-28

**Authors:** L. Barnard, A. M. Portas, S. L. Gray, R. G. Harrison

**Affiliations:** Department of Meteorology, University of Reading, Earley Gate, PO Box 243, Reading RG6 6BB, UK

**Keywords:** eclipse, meteorology, citizen science

## Abstract

The National Eclipse Weather Experiment (NEWEx) was a citizen science project designed to assess the effects of the 20 March 2015 partial solar eclipse on the weather over the United Kingdom (UK). NEWEx had two principal objectives: to provide a spatial network of meteorological observations across the UK to aid the investigation of eclipse-induced weather changes, and to develop a nationwide public engagement activity-based participation of citizen scientists. In total, NEWEx collected 15 606 observations of air temperature, cloudiness and wind speed and direction from 309 locations across the UK, over a 3 h window spanning the eclipse period. The headline results were processed in near real time, immediately published online, and featured in UK national press articles on the day of the eclipse. Here, we describe the technical development of NEWEx and how the observations provided by the citizen scientists were analysed. By comparing the results of the NEWEx analyses with results from other investigations of the same eclipse using different observational networks, including measurements from the University of Reading’s Atmospheric Observatory, we demonstrate that NEWEx provided a fair representation of the change in the UK meteorological conditions throughout the eclipse. Despite the simplicity of the approach adopted, robust reductions in both temperature and wind speed during the eclipse were observed.

This article is part of the themed issue ‘Atmospheric effects of solar eclipses stimulated by the 2015 UK eclipse’.

## 1. Introduction

On 20 March 2015, a partial solar eclipse was observed throughout the United Kingdom (UK). The magnitude of the partial eclipse varied across the UK, from approximately 85% in southern regions, up to approximately 95% in the north. Over the UK, the times of first contact, eclipse maximum and fourth contact were approximately 0830 UT, 0930 UT and 1040 UT, respectively (throughout this article all times are in UT). Atmospheric observations during prior eclipses have demonstrated that the prevailing meteorology can be perturbed by the rapid changes in insolation associated with the propagation of the eclipse penumbra through the atmosphere [1]. In fact, it is known that eclipses significantly affect each layer of the Earth’s atmosphere, from the troposphere to the ionosphere, and a large volume of research has been published on this subject, as reviewed elsewhere in this issue by Aplin *et al*. [2]. Focusing on eclipse-driven meteorological changes, the two most pronounced and readily understood effects are the large decrease in short-wave solar radiation, which can be almost 100%^1^ for total eclipses [3], and a corresponding decrease in air temperature, which can be several kelvin. However, there are also more subtle and complex features in the meteorological response. For example, studies have reported both temporary decreases in the wind speed and anticlockwise rotations in the wind direction [1,4,5]. The dynamical processes driving the observed changes in the wind field have yet to be fully understood and continue to be investigated; see paper by Gray & Harrison in this issue [6]. The infrequent and transient nature of eclipses makes it challenging to obtain sufficient observations to isolate the eclipse-induced changes from the natural evolution of the prevailing weather. Consequently, Harrison and co-workers [1,5] argued that a denser spatial network of meteorological observations could help resolve the atmospheric response and hence lead to an improved physical understanding of eclipse-induced weather changes.

The National Eclipse Weather Experiment (NEWEx) was conceived as a means by which this denser network of observations could be produced, by engaging the efforts of citizen scientists across the UK during the 20 March 2015 partial solar eclipse. For NEWEx, citizen scientists would record simple observations of meteorological parameters throughout the eclipse and submit these via the Internet. These observations would be collated, analysed in the context of the eclipse and the results reported back to the citizen scientists. In doing so, NEWEx would also serve as a valuable public engagement activity, and this aspect of the project is discussed by Portas *et al*. elsewhere in this issue [7]. Previous studies have demonstrated the potential value of engaging citizen scientists in quantitative meteorological research, for example, the UK Citizen Rainfall Network [8] and the Royal Meteorological Societies Big Urban Heat Island project [9]. Temperature changes associated with urban heat islands are approximately several kelvin [10], which is similar to eclipse-driven temperature changes. Therefore, the fact that urban heat island temperature changes have been detected by a citizen science project [9] gives us confidence that a similar approach can be used to detect eclipse-driven temperature changes.

In §2, we describe the objectives, design and development of NEWEx. Section 3 describes the data processing. In §4a, the accuracy of an example of the NEWEx temperature data is assessed by comparison to a well-established record of air temperature measurements taken at the University of Reading’s Atmospheric Observatory. Sections 4b–d present the nationwide results of the NEWEx analysis for temperature, cloudiness, and wind speed and direction. Our conclusions on the outcomes of NEWEx are summarized in §5.

## 2. Objectives and design

### (a) Objectives and design criteria

NEWEx had two principal objectives:

(i) To use citizen science to aid the investigation of solar eclipse effects on meteorology. More specifically, to provide a spatially dense network of regular meteorological observations across the UK and hence meet a need identified by previous research [1,5].
(ii) To develop a nationwide public engagement activity for the eclipse based on the participation of citizen scientists in NEWEx.

On the basis of prior studies [1,5], it was decided that participants would be asked to report observations of the air temperature, cloud coverage, and wind speed and direction, which are important meteorological variables [11]. Consideration of these objectives led to two design criteria that NEWEx needed to fulfil. Firstly, as NEWEx would ask participants to make multiple observations of up to four parameters, it was necessary that these observations be practically achievable for those unfamiliar with weather measurements, and also that a simple and efficient means was provided to report the observations to NEWEx. Secondly, NEWEx would be more successful from a public engagement perspective if the results were communicated back to the participants quickly and clearly, immediately demonstrating the value in their contributions. Therefore, it was necessary that the NEWEx analysis could be completed quickly. It was decided to attempt to process the NEWEx data in close to real time and that, to achieve this, all of the required analysis should be prepared and automated in advance, such that during the eclipse our efforts could be focused on reporting the results.

The Internet clearly provides the simplest and most efficient means for participants to report their results, although there are a large variety of ways through which such a system could be implemented. Given the resources available to NEWEx, a Google Form provided the best option for data collection, as it was free, easy to configure and could certainly handle the total volume and throughput of submissions that NEWEx could reasonably expect to receive. The design and development of the NEWEx webform are discussed in an accompanying publication in this issue [7]. The core of the NEWEx processing system was developed in the Python programming language, as it has all the necessary functionality to interface with the Google Form to access the data, perform the data analysis, and produce and upload the graphical outputs to the Internet. Prior to 20 March, this system was tested with synthetic submissions to the webform, such that we were confident that NEWEx would operate smoothly and in close to real time during the eclipse.

In developing the webform, it was decided that it should be designed so that a participant could report all their observations over the eclipse period in one submission. In comparison to providing a submission for each round of observations, this would reduce the labour required by the participants and make engagement with NEWEx more achievable. After several design iterations, the parameters to be observed were discretized into classification windows, as were the observation times, such that multiple observations could be submitted via a matrix of radio buttons for each parameter (see section 2b and fig. 1 in [7]). We now briefly describe the scale and range that were used to classify each parameter.

### (b) Time

NEWEx asked participants to collect observations between 0800 and 1100, and these were split into 21 observation windows, consisting of a higher resolution period (5 min observations) during the main phase of the eclipse (0900–1000), and two lower resolution periods (15 min observations) before and after this (0800–0900 and 1000–1100). Rather than being directed to coordinate their observations with these set times, participants were asked to select the observation window that best matched the time of their observations. The 0800–1100 window was chosen as it would provide the important observations before and after first and fourth contact, respectively, such that weather changes associated with the eclipse could be separated from the background weather. The number of times available for entries was chosen to strike a balance between the desire for high-frequency observations, which would make it easier to resolve eclipse-induced changes, and those which could practically be achieved by manual observers. The observation window was split into periods with different temporal resolutions, as it was expected that the transient features associated with the main phase of the eclipse would evolve more quickly than the background weather; for this period it would be beneficial to increase the observation frequency.

### (c) Temperature

Temperatures were reported in 0.5°C increments from −10°C to 20°C. The range was chosen by considering the climatology for the UK, across a typical range of altitudes. Near to the event time, this range was changed a little based on the long-range forecast (doing this on the basis of a short-range forecast might well have helped things). The temperature resolution used was chosen as a compromise between the magnitude of the expected temperature changes, the typical accuracy and precision of common thermometers, and the range of temperatures that needed to be covered. For example, although it is clearly beneficial to cover a wide range of parameter values at high resolution, including too many values would make the webform interface cumbersome. Analysis of the NEWEx observations suggested this was an infrequent problem, but there are suggestions that the inclusion of sub-zero temperatures may have caused some confusion. For example, one submission was found to have submitted temperatures very similar in magnitude to several other closely located submissions, except that the temperature values entered were negative.

Participants were provided with basic instructions on how to measure air temperatures, stating that measurements should be taken in the shade to reduce the effect of radiation errors [12]. The type of thermometer that should be used was not specified, other than requiring a resolution of at least 0.5°C. This decision was made to help encourage participation, as it was considered that overly specifying the type of thermometer could make NEWEx inaccessible to some groups. This means that we are unable to quantify the errors associated with each submitted temperature. Meteorological thermometry was reviewed by Harrison [12], who concluded that the accuracy of liquid-in-glass thermometers is typically ±0.2°C whereas the accuracy of some inexpensive digital thermometers can be much poorer. These errors are comparable in magnitude to the eclipse-induced temperature effects that we aim to observe. However, by averaging multiple submissions, these errors can be suppressed and robust estimates of temperature can be made; for this reason, NEWEx relies on effective participation. Therefore, we can have more confidence in the NEWEx results in regions with high participation, while the uncertainties are larger in areas with lower participation.

### (d) Cloudiness

Measurement of cloud is something for which some training of an observer is known to be useful. Conventional meteorological practice is to record both the level of the cloud (as low, medium and high), and the cloud coverage at each level, in eighths of the sky covered. For this project, a much simplified approach was used. Cloudiness observations were divided into the four categories of clear sky, some cloud, much cloud and totally overcast.

### (e) Wind speed and direction

Accurate wind speed measurements require an anemometer, but this was not an instrument considered likely to be available to most of the participants. Wind speed can, however, be estimated entirely without instrumentation from its visible effects, using the Beaufort system, and this was the approach adopted. Wind speeds were assessed and reported in terms of the Beaufort force and the standard descriptions of each force, between Forces 0 and 6. Wind directions were classified by visual estimation of direction, using the eight-point compass directions: north, northeast, east, southeast, south, southwest, west and northwest.

Using simple visual assessments of the cloudiness and wind speed and direction has some advantages. Most importantly it meant that the only apparatus required to participate in NEWEx was a timepiece and a basic thermometer. This, coupled with the simplicity of the observations, fulfils the requirement that the observations be practically achievable for participants with a wide range of resources and abilities. However, a disadvantage of requesting such low-fidelity observations was that there was uncertainty about whether they would be able to resolve the small meteorological changes resulting from an eclipse. Clearly, this represented a compromise between encouraging participation with the minimum level of instrumentation, and the limited resolution and accuracy provided by the estimations made.

### (f) Location

It was necessary to designate a location to each of the observations submitted via the NEWEx webform. In the design stage of NEWEx, it was decided that participants would be required to provide their location using the postcode of where the observations were made. Postcodes in the UK are an alphanumeric code used by the postal service to designate geographical delivery areas. The basis for this decision is that we considered it likely that participants would have access to an appropriate postcode, but may not know their latitude and longitude, or how to access this information conveniently or reliably.

A complete postcode is referred to as a ‘postcode unit’ and each unit consists of a ‘sector’, ‘district’ and ‘area’, which isolate progressively larger geographical areas. For example, the postcode unit of the Meteorology Department at the University of Reading is RG6 6BB. This unit has the following components: the postcode area is RG, for the Reading postal office; the postcode district is RG6, for the Earley suburb of Reading; and the postcode sector is RG6 6, which isolates mainly the portion of Earley spanned by the University of Reading Whiteknights campus.

To convert the postcodes into physical coordinates, the Ordnance Survey Code Point Open dataset was used (https://www.ordnancesurvey.co.uk/business-and-government/products/code-point-open.html). Code Point Open is a freely available database that provides the easting and northing coordinates of the approximately 1.8 million postcode units used by the UK’s Royal Mail. The conversion from easting and northing to latitude and longitude was facilitated by the ‘oscodepoint’ Python package (https://pypi.python.org/pypi/oscodepoint). Using the Ordnance Survey codepoint data and the oscodepoint Python package, it is possible to algorithmically convert postcodes into latitude and longitude coordinates.

Obtaining this location data is critical to the subsequent analysis, and so it was necessary to consider how to process incomplete or invalid postcode units. It was decided to also calculate approximate coordinates for the locations more coarsely defined by the postcode districts and postcode areas. To do this, we calculated the average latitude and longitude of the set of postcode units in their respective districts and areas. This provided, in effect, an average coordinate weighted approximately by the population density in that region. If a postcode unit submitted by a participant could not be matched to a valid postcode unit, we tried to associate it with a valid postcode district or, failing that, a postcode area. If no geographical location could be attributed to the observations, they were not analysed further. We are unable to check for postcodes that were valid but incorrectly entered, which is a source of error that we cannot quantify.

## 3. Data processing

### (a) Pre-analysis processing

**Figure 1.**
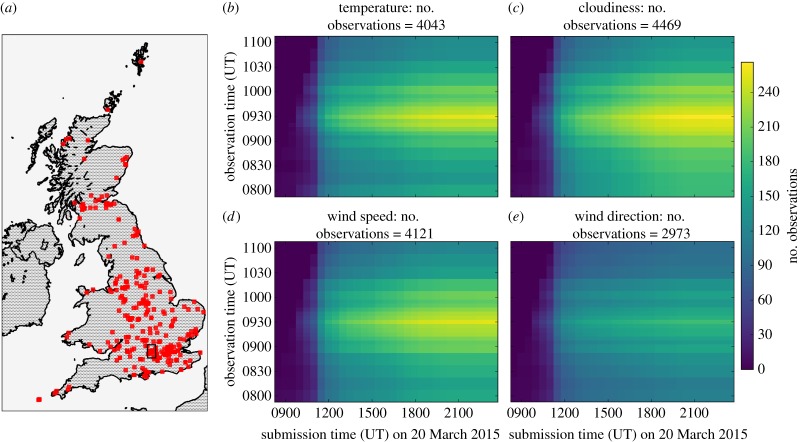
(*a*) Map of the UK with the red squares marking the locations of individual NEWEx submissions that were found to have some valid data. The black box marks the 0.2°×0.2° region centred on the University of Reading’s Atmospheric Observatory, which is analysed in §4a. (*b*–*e*) The number of observations for each of the available NEWEx observation times as a function of the time that these data were submitted to NEWEx, for temperature, cloudiness, wind speed and wind direction, respectively. These four panels all share common axes and scaling.

Several phases of quality control and processing were applied to the data before they were used in any analysis. In total, NEWEx received 503 submissions via the webform, of which 15 were removed as they were submitted before the start time of the experiment (0800) and so could contain no valid data. Of the remaining 488 entries, 40 provided location information in a format other than a standard UK postcode. From the information provided, we established full or partial UK postcodes for 23 of these 40, and the remaining 17 were removed, including three entries from The Netherlands, Spain and the Republic of Ireland. Therefore geographical coordinates were established for 471 entries, with 420 linked to a complete postcode unit, 47 to a postcode district and 4 to a postcode area. These 471 entries correspond to 309 unique locations, as several participating groups made multiple submissions. As the overwhelming majority of submissions were linked to a full postcode unit, hereafter it is assumed that any errors associated with the approximate locations attributed to the postcode districts and areas will have a negligible effect on our analyses. The geographical distribution of NEWEx submissions is illustrated in figure 1*a*, demonstrating that the submissions extend across the southern and central regions of the UK but become increasingly sparse to the northern and western regions, appearing to approximately follow the distribution of population density. Many submissions are clustered around London and in southeast England.

Further to removing submissions received before the beginning of the experiment, it was also considered prudent to remove all measurements attributed to observation times later than the recorded submission time. In these instances, we do not remove the entire submission, but mask the erroneous observations from further analysis. This error occurred infrequently and removes only 41 values from the complete set of 15 606 observations collated by NEWEx.

Figure 1*a* highlights that the NEWEx observations are not uniformly spatially distributed, while figure 1(*b*–*e*) considers how the observations are distributed among the NEWEx observation times and as a function of submission time. For each parameter and each observation time, the number of observations received by NEWEx before a given submission time was calculated for submission times ranging from 0830 until 2330, in steps of 30 min. The distribution of the number of observations is shown for temperature, cloudiness, wind speed and wind direction in figure 1*b*,*c*,*d* and *e*, respectively. Each panel uses the same scaling given by the colour bar on the right of the figure.

One objective of NEWEx was to analyse submissions in close to real time and to report online the findings as the eclipse progressed. Figure 1*b*–*e* demonstrates why this objective was difficult to achieve on the day. Very few submissions were received during the main phase of the eclipse, with a drastic rise in the number of submissions after 1100. The submissions continue throughout the day, but, for each parameter, the distribution of the number of observations across the observation times has approximately converged to its final shape by 1900 onwards. Ideally, these distributions would be uniform, with approximately equal numbers of observations at each observation time. However, there was clearly a strong behavioural preference for participants to provide observations during the main phase of the eclipse, with the periods before and after having approximately half the number of observations.

Comparing the distributions across the parameters demonstrates that, while the temperature, cloudiness and wind speed parameters have a similar number of observations (4043, 4469 and 4121, respectively), there are significantly fewer wind direction observations (2973). It is unclear exactly why this is. Wind speed was typically quite light on the day, which may have made assessing the wind direction more difficult, and many of the NEWEx observations were recorded in an urban environment, which may have also complicated assessing the wind direction [13].

### (b) Gridding

**Figure 2.**
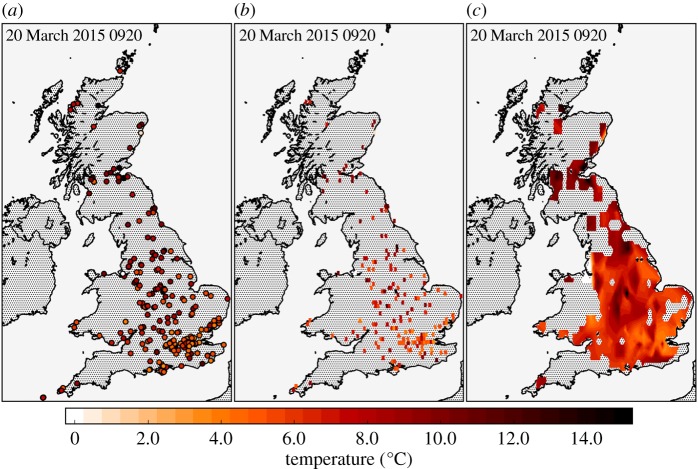
Panels demonstrating the gridding process using the NEWEx temperature observations at 0920. (*a*) Markers show locations of NEWEx submissions providing temperature observations at 0920, where markers are coloured according to the observed temperature. (*b*) Grid cell means of the NEWEx temperatures. (*c*) Bi-linearly interpolated, masked and contoured temperature field.

To analyse the spatial evolution of the observed meteorological fields, they were gridded onto a 0.1°×0.1° latitude–longitude grid, spanning from −8.2° to 2.0° longitude and 49.0° to 60.4° latitude. For each field and each observation time, the mean of the NEWEx observations in each grid cell was calculated. Bi-linear interpolation was used to fill in grid cells with no data. In the case of the cloudiness observations, the clear sky, some cloud, much cloud and totally overcast categories were converted into 0, 1, 2 and 3, respectively. As shown in figure 1*a*, the observation network has regions that are sparsely populated, where the bi-linear interpolation may yield estimates that are poorly constrained due to being based on widely separated observations. To limit this, we apply a mask to the interpolated fields, removing all interpolated values that are five or more grid cells from a cell containing NEWEx observations. The gridding process is demonstrated graphically in figure 2.

### (c) Temperature anomalies

The eclipse occurred during the morning, when near-surface air temperatures tend to increase as part of the diurnal cycle. This diurnal signal can complicate identifying an eclipse-driven response in near-surface air temperatures. Therefore, for each grid cell, we also calculate a temperature anomaly, calculating the difference between the observed temperatures and an estimate of how the temperature would have evolved without the eclipse. The expected temperature evolution without the eclipse is estimated by assuming that the diurnal rise in temperatures is approximately linear over the period of the eclipse. The ordinary least-squares regression of the 0800–0830 and 1030–1100 temperature observations against observation time provides the linear fit used to estimate the expected air temperature without the eclipse, at times between first contact and fourth contact. With this fit, the temperature anomaly is calculated at each observation time throughout the eclipse. Figure 1*b* clearly shows that there are fewer observations in the 0800–0830 and 1030–1100 periods than during the eclipse. The result of this is that there is a fraction of grid cells where no temperature anomalies can be calculated because of insufficient data coverage. Therefore, the temperature anomaly field has sparser spatial coverage than the temperature field.

## 4. Results

### (a) Assessing the accuracy of NEWEx temperature estimates

We now consider how accurate the NEWEx temperature estimates may be, by comparing them against a long-established source of reliable near-surface air temperature observations taken from the University of Reading’s Atmospheric Observatory (URAO) (http://www.met.reading.ac.uk/observatorymain/index.html). The URAO is located at a latitude of 51.441° N, longitude of 0.938° W, and an altitude 66 m above mean sea level. Air temperature observations were made with a platinum resistance thermometer situated 1.25 m above ground, housed inside a Stevenson screen, and recorded with a frequency of 1 Hz. The Stevenson screen acts to protect and isolate the thermometer from factors that could cause errors in the air temperature measurements, such as direct solar radiation and precipitation [12]. We use 1 min means of the URAO air temperature measurements, as provided in the electronic supplementary material of Burt [14] in this issue, which also includes further details of the observatory and discussion of the eclipse effects on the weather at URAO.

The temperature time series for this area was also calculated from the NEWEx observations. All submissions within a 0.2°×0.2° latitude–longitude box centred on the coordinates of the URAO were identified. This region is marked by the black box in figure 1*a*. There were 15 submissions that provided temperature estimates in this region, although one was removed from further analysis as the participant provided only one temperature observation of 17°C at 0920, which was clearly an outlier. For each NEWEx observation time, the mean of the temperature estimates was calculated.

**Figure 3.**
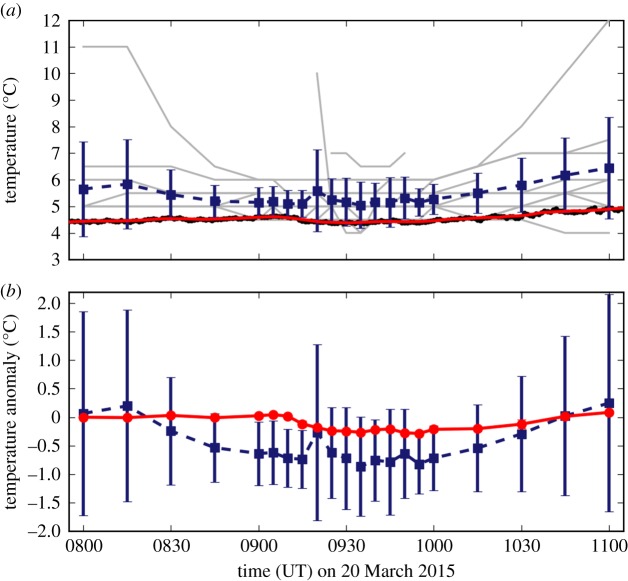
(*a*) Time series of the temperature estimates for the Reading region, marked by the black box in figure 1*a*. The black dots show the 1 min mean temperatures from the URAO, while the red line is a 15 min rolling window mean of these data. The grey lines show the sequences of temperature observations for each submission. The blue squares show the mean of the NEWEx temperatures at each observation time, while the error bars are two standard errors of the mean. (*b*) Time series of the temperature anomaly for the NEWEx and URAO temperature series, in blue (squares) and red (dots), respectively.

Figure 3*a* shows the NEWEx and URAO temperature observations. The comparison is demanding as, due to the layer of cloud present in the Reading area throughout the eclipse, the temperature variation was considerably suppressed over that expected in clear skies. Grey lines show the temperature time series for individual NEWEx submissions. Note that fewer than 14 submissions are visible, as the discrete nature of the submitted temperatures, and agreement between observers, means several lines overlap. The blue line shows the mean of the NEWEx submissions, where the error bars represent two standard errors of the mean. The black dots mark the 1 min mean URAO temperature time series, while the red line shows the 15 min running boxcar-window mean of these data.

The mean NEWEx temperature series is biased to higher temperatures relative to the URAO series, although the URAO series typically lies within the lower limit of the two standard errors of the mean NEWEx temperature. The bias varies from a minimum of 0.5°C to a maximum of 1.5°C, with a mean value of 0.9°C. Although undesirable in absolute terms, the small bias is considered reasonable for the following reasons: firstly, it is unlikely that the NEWEx temperature observations were made in an environment as effectively exposed as URAO; secondly, it is unlikely that they were taken within an environment designed to minimize errors due to other environmental factors, such as that provided by the Stevenson screen at URAO. The overcast conditions in the Reading region (figure 6) mean that it is unlikely that a radiation error from direct solar heating accounts for much of this difference. Prior research into the differences between measurements of open air temperature and screen temperature demonstrates that for surface insolations of more than 500 W m^−2^ the temperature difference is rarely larger than 0.5°C [15]. The URAO observations show that, due to heavily overcast conditions in the southern UK, global solar radiation at the surface was low throughout the morning, being less than 160 W m^−2^ until 1100 [14]. Therefore, although NEWEx temperature observations are certainly subject to some radiation error, it is very unlikely that this explains much of the observed warm bias. However, it is possible that factors such as observers working in more sheltered and more urban environments, as well as possibly holding the thermometer, may explain this bias. Furthermore, the NEWEx temperature estimate is based on observations over the 0.2°×0.2° area centred on URAO, and the average temperature over this region should be expected to differ somewhat from a point measurement at URAO.

Although there is a systematic difference between the NEWEx and URAO temperatures, it is possible that they both show a similar relative response during the eclipse. This is considered here by comparing the temperature anomaly of both the URAO and mean NEWEx temperature series, which was calculated as described in §3c. As discussed in §2, the NEWEx observation times are split into a higher frequency (5 min) period and two lower frequency (15 min) periods. To make a fair comparison between the mixed resolution NEWEx observations and the uniform URAO observations, the URAO data are processed before the temperature anomaly is calculated, so that they have a mixed resolution similar to NEWEx. For the higher resolution period, 5 min averages of the URAO data are used, while, for the two lower resolution periods, 15 min averages of the URAO data are used. Figure 3*b* shows the NEWEx and URAO temperature anomaly data, in blue and red, respectively. Error bars are included for the URAO temperature series; however, they are sufficiently narrow that they do not appear outside the markers. Both temperature anomaly profiles show a shallow decrease in temperature over the eclipse period. The NEWEx anomaly shows a maximum decrease of 0.86±0.86°C at 0935 while the maximum decrease in the URAO series was 0.29±0.01°C at 0955. Burt [14] also analyses the URAO temperatures during the eclipse and, on the basis of the 1 min mean values, estimates that the maximum temperature anomaly during the eclipse was ≈0.6°C. The magnitude of the eclipse response should be larger in the higher temporal resolution 1 min values analysed in [14], and so we consider these estimates to be consistent. There is a 20 min delay between the maximum anomaly in the NEWEx and URAO temperature series. However, given the very calm conditions, it was estimated in [14] that the URAO temperature observations from within the Stevenson screen would be lagged behind the true air temperature by approximately 5–10 min, and so it is reasonable that the maximum temperature anomaly occurred later in the URAO observations.

Therefore, we conclude that NEWEx performs reasonably well at monitoring the meteorological conditions for a region, as the differences between the NEWEx temperature observations and the well-established URAO observations are small and can be explained in terms of known features of the measurement systems and data processing.

### (b) Eclipse-induced temperature effects

**Figure 4.**
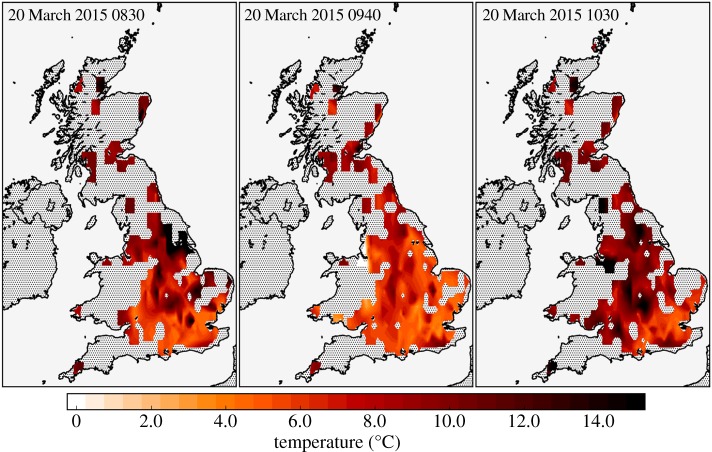
Maps showing contours of the NEWEx temperature fields at 0830, 0940 and 1030. These are the NEWEx observation times that most closely correspond to the times of first contact, maximum temperature anomaly and fourth contact, respectively.

**Figure 5.**
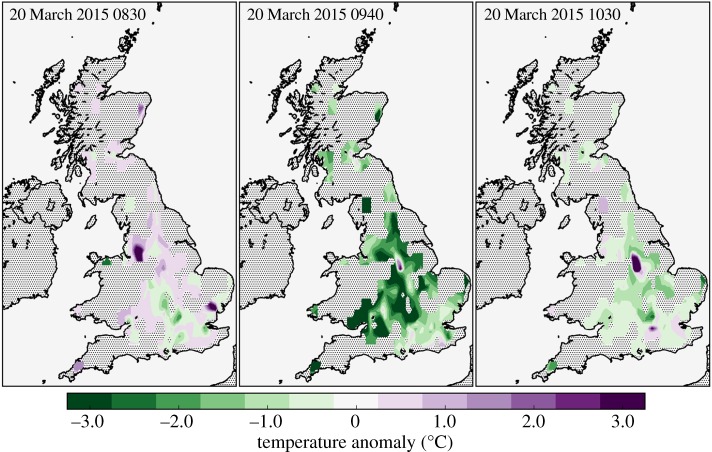
As for figure 4, instead showing contours of the estimated temperature anomaly, calculated as described in §3c.

Figures 4 and 5 show contours of temperature field and temperature anomaly field estimated from the gridded NEWEx observations at 0830, 0940 and 1030. The times 0830 and 1030 are the closest observation times corresponding to the times of first contact and fourth contact, respectively, and 0940 is the mean time when, according to the NEWEx data, UK sites experienced a maximum temperature anomaly. The mean of the maximum temperature anomalies was −2.2 ± 0.3°C.

**Figure 6.**
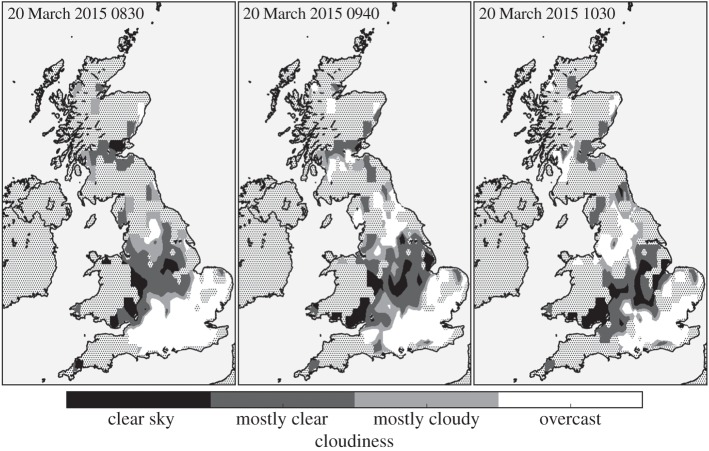
As for figure 4, instead showing contours of the cloudiness observations.

There was no apparent relationship between the time of the maximum temperature anomaly and the geographical location, as might be expected from the approximately northeastward propagation of the eclipse over the UK, with the times of maximum obscuration varying from approximately 0925 in the UK’s southwest peninsula to 0940 in the northwest of Scotland. However, this may be due to the 5 min resolution of the temperature observations being too low to resolve any such relationship, and because the majority of the NEWEx submissions were clustered in the central and southeastern regions, where the maximum obscuration times were similar, occurring at approximately 0930. As the mean time of the maximum temperature anomaly was 0940, this implies that the lag between maximum obscuration and maximum temperature anomaly was typically about 10 min. Comparing the structure of the temperature anomaly fields (figure 5) with the cloudiness fields (figure 6) shows that, as expected, temperature anomalies are smaller in magnitude in regions of increased cloudiness.

Elsewhere in this issue, Clark [16] analyses 1 min resolution temperature observations from 266 sites contributing to the UK Met Office’s Meteorological Monitoring System (MMS) and finds eclipse-induced temperature anomalies that range between −0.03°C and −4.23°C, with a median value of −1.02°C. The temperature anomalies derived from the NEWEx observations are broadly consistent with this, as figure 5 shows that the 0940 temperature anomalies are typically within the range observed in [16]. Furthermore, there is fair agreement between the structure of the temperature anomaly field at 0940 and the contours of the maximum temperature anomaly presented in fig. 7*a* of [16]. However, we do note that Clark [16] reports a smaller average temperature anomaly and a longer average lag (≈15 min) between maximum obscuration and the maximum temperature anomaly. These differences are similar to those found when comparing the NEWEx and URAO temperature anomalies. The MMS temperature data are derived from 266 locations, whereas the NEWEx data come from 309 locations, but the spatial distributions of these networks are very different: MMS has a more even coverage, being less clustered around population centres. Furthermore, the MMS temperature measurements are recorded within Stevenson screens, which, similar to the comparison with URAO data, may partly explain the difference in the observed lag between maximum obscuration and maximum temperature anomaly.

**Figure 7.**
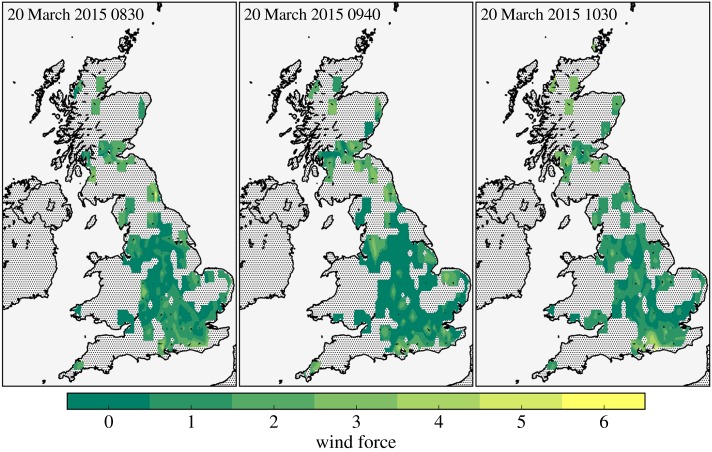
As for figure 4, instead showing contours of the wind speed.

### (c) Eclipse-induced effects on cloudiness

Figure 6 shows contours of the cloudiness at 0830, 0940 and 1030. These reveal that much of the southeast of the UK was overcast throughout the eclipse, with a band of mostly clear and cloud-free conditions across the Midlands. This is in good agreement with both the visible and infrared remote sensing images of the cloud field during the eclipse (cf. fig. 7*a* of [16] and fig. 1*b* of [6]) and, as discussed in §4b, is consistent with the observed temperature anomalies.

Resolving changes in cloudiness caused by the eclipse is an important practical question, as images of the eclipsed Sun are scientifically very useful within the solar physics and astronomy communities (see, for example, [17,18] and Scott *et al*. [19] in this issue), and there is enormous public interest in viewing the eclipse. Therefore, it is important to determine whether or not the eclipse can cause changes in the cloud field that could make a successful eclipse observation more or less likely. From figure 6, there is a suggestion that the region of mostly clear and clear sky was broader at 0940 than at either 0830 or 1030, which could perhaps be an eclipse-driven change in cloudiness. However, there were also weather-related changes in cloud during the same interval, due to the slow southwards passage of a weak weather front, which complicates attributing changes in the cloud field to eclipse-driven effects.

A sequence of *χ*^2^-tests of homogeneity was employed to infer whether the data suggest that the amount of cloudiness changed between 0830, 0940 and 1030. Only grid cells containing NEWEx observations at each of 0830, 0940 and 1030 were analysed. Therefore, no interpolated data are analysed and the spatial distribution of the observations used in the statistical tests does not change. For these grid cells, the histogram of the cloudiness observations was calculated for each time and these data are compiled into the contingency table in table 1; note that the total count is the same at each time due to the requirement that each grid cell include observations at each of 0830, 0940 and 1030. It is immediately clear from table 1 that the histograms of cloudiness are very similar for each time. The *χ*^2^-test of homogeneity is used to assess whether the observed proportions of cloudiness observations are consistent with the null hypothesis that they are time-independent, i.e. that the distributions of cloudiness did not change with time. The test is implemented as described in [20] and compares the observed occurrence with the expected occurrence under the null hypothesis of homogeneity. More details on the statistical testing procedures are given in appendix A. This test was employed on each of the three unique pairings of the cloudiness distributions at 0830, 0940 and 1030, i.e. 0830–0940, 0830–1030 and 0940–1030. The *χ*^2^-values and *p*-values of these three tests are included in table 2. For a single statistical test, it would be common to compare these *p*-values against a criterion of *p*<0.05, which would indicate that the data were unlikely to have been obtained under the null hypothesis. However, here, multiple comparisons are performed and it is appropriate to use a Bonferroni-adjusted critical *p*-value, such that the appropriate criterion is *p*<0.017. These *p*-values are much larger than the Bonferroni-adjusted critical *p*-value, which indicates that these data are not inconsistent with the null hypothesis that there was no change in these cloudiness distributions with time. Therefore, these tests provide no evidence that there was an eclipse-induced response in the cloud field. We stress that this lack of a significant change does not mean that there is no response in the cloud field to the eclipse. Instead, the low fidelity of the cloudiness observations and changes in the data coverage across the NEWEx observation period mean that we are unable to draw any definitive conclusions about whether or not there were eclipse-driven changes in the cloud field. Although a more sensitive determination of cloudiness may have been beneficial, it is necessary to bear in mind the importance of the measurements being practically achievable for a wide range of abilities; a more sensitive measure of cloudiness, such as resolving cloud amount in eighths (oktas) of sky coverage, may have also been more difficult for participants to assess, and so could have led to fewer reliable observations.

**Table 1.** Distributions of cloudiness before, during and after the eclipse.

time (UT)	clear sky	mostly clear	mostly cloudy	overcast	total
0830	17 (12%)	39 (28%)	28 (20%)	56 (40%)	140
0940	18 (13%)	37 (26%)	31 (22%)	54 (39%)	140
1030	21 (15%)	37 (26%)	30 (21%)	52 (37%)	140

**Table 2.** Results of the *χ*^2^ homogeneity tests on distributions of cloudiness.

comparison	*χ*^2^	*p*-value
0830–0940	0.27	0.966
0830–1030	0.69	0.875
0940–1030	0.28	0.963

### (d) Eclipse-induced effects on wind speed and direction

Figure 7 shows contours of the NEWEx wind speeds at 0830, 0940 and 1030, in terms of the Beaufort wind force. Winds were typically very light across the UK throughout the eclipse period. The limited spatial coverage and low fidelity of the wind speed observations also make it difficult to interpret the estimated wind speed fields. However, figure 7 does appear to show limited evidence of an increased occurrence of weaker wind speeds at 0940 relative to 0830 and 1030.

The same *χ*^2^ statistical testing procedure that was used with the cloudiness observations was also employed with these wind speed data. Again, only grid cells containing wind speed observations at each of 0830, 0940 and 1030 were analysed, such that no interpolated data were analysed and the spatial distribution of the observations is the same at each time. The histograms of the wind force observations are compiled into the contingency table given in table 3; wind speeds greater than Force 3 were merged into a Force ≥3 category, to meet the recommendation of the *χ*^2^-test of homogeneity that no categories have fewer than five counts. A clear feature of the values in table 3 is a marked increase in the occurrence of Force 0 winds at 0940 relative to 0830 and 1030, as well as a similar decrease in Force 2 winds. This is suggestive of a decrease in reported wind speeds at 0940. For these wind speed data, the *χ*^2^-values and *p*-values are reported in table 4. These *p*-values do not meet the Bonferroni-adjusted *p*-value criterion of *p*<0.017 that would indicate that these data are unlikely to have been obtained under the null hypothesis that the wind speed distributions were the same at all times. However, the 0940–1030 comparison is close to the critical value, which suggests that these data are moderately inconsistent with the null hypothesis. As recommended in [20], it can be instructive to look at such data in more detail. Here, this is done by computing the standardized residuals between the observed occurrence and expected occurrence under the null hypothesis of homogeneity, using the method given in [20], which is described in appendix A. The results are *p*-values for each cell of the 2×4 contingency table for the 0940–1030 comparison, which are reported in table 5. For the commonly used test level of *α*=0.05 (see appendix A), these *p*-values should be compared against a Bonferroni-adjusted *p*-value of *p*<0.006. Table 5 shows that the *p*-values of the Force 0 wind speeds are close to (but just larger than, at the fifth decimal place) the critical value, suggesting that these values are quite unlikely to have been obtained under the null hypothesis of homogeneity. Considering these statistical tests in conjunction with the observed increase in the occurrence of reported Force 0 wind speeds at 0940 suggests that there is reasonable evidence that the NEWEx observations reveal a temporary slight decrease in the wind speed near the time of the maximum temperature anomaly. Gray & Harrison [6] used the UK Met Office’s MIDAS observation network and another network of roadside observations operated by Vaisala to assess wind changes associated with the 20 March 2015 eclipse, and found a decrease in the wind speed of up to 2 knots. The analysis of the NEWEx results appears to be consistent with this.

**Table 3.** Distributions of wind speed before, during and after the eclipse.

time (UT)	Force 0	Force 1	Force 2	Force ≥3	total
0830	19 (21%)	38 (42%)	26 (29%)	7 (8%)	90
0940	31 (34%)	32 (36%)	18 (20%)	9 (10%)	90
1030	15 (17%)	36 (40%)	29 (32%)	10 (11%)	90

**Table 4.** Results of the *χ*^2^ homogeneity tests on distributions of wind speed.

comparison	*χ*^2^	*p*-value
0830–0940	5.10	0.165
0830–1030	1.21	0.748
0940–1030	8.43	0.038

**Table 5.** The *p*-values of wind speed standardized residuals before, during and after the eclipse.

time (UT)	Force 0	Force 1	Force 2	Force ≥3
0940	0.006	0.539	0.062	0.808
1030	0.006	0.539	0.062	0.808

Assessment of the wind direction appears to have been challenging, as indicated by the comparatively fewer wind direction observations relative to the temperature, cloudiness and wind speed observations (figure 1). As of yet we have been unable to establish if the reported wind directions can be used to infer anything about eclipse-driven changes in the wind direction. Gray & Harrison [6] also investigated eclipse-driven wind direction changes and found a temporary backing of the wind direction by approximately 20°. These direction changes are smaller than could easily be resolved by the NEWEx wind directions, which were recorded in terms of the eight-point compass directions, and so have an angular resolution of 45°. The low angular resolution of the NEWEx wind directions, coupled with slightly less participation in the wind direction measurements, means that it may be difficult to glean any information on eclipse-driven wind direction changes from the NEWEx data.

## 5. Conclusion

The NEWEx was, as far as we know, a world first, in measuring and analysing eclipse changes in the weather on a national scale, in close to real time, through engagement of a network of citizen scientists. Through this engagement with the public, NEWEx collected 15 606 meteorological observations from 309 locations within the UK. From these data, we have been able to derive estimates of the near-surface air temperature, cloudiness and near-surface wind speed fields across many UK sites. Additionally, NEWEx gained the attention of both the national and local press and was well received by the public, and it therefore served as a very positive science outreach experience. These aspects of NEWEx are discussed in [7].

The estimates of the near-surface air temperatures and eclipse-driven temperature anomalies were consistent with other well-established means of measuring these parameters, such as with measurements from the URAO and the UK Met Office’s MMS. This is quite remarkable given the simplicity of the observations required by participants in NEWEx and the small magnitude of the changes observed.

The low fidelity of the wind speed, wind direction and cloudiness observations provided a broad picture of these conditions over the UK, but did make it difficult to detect eclipse-driven changes in these fields. Statistical tests were employed to assess whether there were differences between the distributions of both cloudiness and wind speed at times just before the eclipse, at the mean time of the maximum temperature anomaly, and at the end of the eclipse. There was no evidence to suggest that the distribution of reported cloudiness changed over these periods. However, there was reasonable evidence to suggest a temporary decrease in the reported wind speed, which was shown to be consistent with the analyses of professional meteorological data of this eclipse [6].

Analysis of the submitted observations demonstrated comparatively fewer wind direction observations relative to temperature, cloudiness and wind speed. This suggests that participants found it difficult to estimate the wind direction. This could be improved upon by suggesting that participants use a compass and some kind of tracer to estimate the wind direction, for example, soap bubbles or blades of grass. The data collection system for NEWEx worked effectively, but, given its critical role in the success of NEWEx, it is worth considering how it could have been improved. Of the 488 submissions, 40 provided location data in a format other than the requested postcode format. The impact of these errors appears to have been small, as approximate locations were obtained for 23 of these 40, while the remaining 17 were removed. Changes to the structure of the NEWEx webform could have potentially avoided this error. For example, a validation rule could have been used to return errors to participants submitting postcodes that do not conform to known properties of postcodes (for example, postcodes have a strict minimum and maximum possible length and should be alphanumeric). This functionality is available within the Google Form service used by NEWEx. Furthermore, although we could identify and remove invalid postcodes, we were unable to identify submissions that used a valid but inappropriate postcode. The occurrence of this error could be reduced by including a dynamic map on the NEWEx webform, which identifies the location of a participant’s submitted postcode, such that the participant can validate their own submission. Finally, in the pre-analysis processing of the NEWEx data, it was established that a small fraction (0.2%) of data was submitted at times prior to the recorded observation time, which is clearly an error. This error could be avoided by developing a dynamic webform that updates throughout the experiment, to limit the possibilities for participants to submit data in error. However, implementing the suggested solutions to the identified limitations would have required significantly more resources in the development stage of NEWEx, probably requiring a custom-made webform; this was beyond the scope of the resources available to NEWEx, but could be worth considering for future studies employing a similar method.

The level of participation in NEWEx was good, with submissions being received from across the UK. This was in part achieved by liaising with appropriate institutions, such as the South-East Physics Network, the Scottish Institute of Physics, the Royal Society of Edinburgh and the BBC. This was supplemented by advertising NEWEx on both local and national radio, as well as social media engagement via Twitter. Participation was higher in urban areas, and in particular the southeast, which is probably due to increased population density. NEWEx could have been improved with more participation from rural and remote areas; this could possibly be achieved by directly targeting rural schools.

Considering these results, it is clear that NEWEx achieved both of its principal objectives, having provided a spatial network of meteorological observations that detected eclipse-driven weather changes and also a successful nationwide public engagement activity. In August 2017, a total solar eclipse will be visible from North America, propagating from the east coast to the west coast of the USA, providing another opportunity to study eclipse-induced meteorology changes. NEWEx serves as a useful example of the strengths and challenges of using a citizen science approach to study eclipse-induced meteorological changes, and could provide a template for a similar study for the August 2017 eclipse.

## Footnotes

1 A small fraction of solar short-wave radiation received at Earth is emitted from the solar corona, which is never fully eclipsed.

## Data accessibility

The National Eclipse Weather Experiment observations analysed in this paper are available from the University of Reading Research Data Archive at http://dx.doi.org/10.17864/1947.68.

## Appendix A

**(a) Bonferroni-adjusted *p*-values**

In null hypothesis significance testing, it is common to calculate some test statistic, for example a *χ*^2^-value, and to compare this with the distribution of values of the test statistic that would be expected if the null hypothesis were true. If the observed test statistic is sufficiently unlikely to occur under the null distribution, this indicates that these data are unlikely to have been obtained if the null hypothesis were true [21]; this can be interpreted as evidence against the null hypothesis [20]. Here, ‘sufficiently unlikely’ is a subjective judgement that must be made by the investigators, although it is typical to set a criterion of *p*<*α*, where *p* is the probability of obtaining a test statistic at least as improbable as the observed value, and *α* is the ‘level’ of the test, commonly set at *α*=0.05 [21]. A Type-I error, or false-positive, occurs when the null hypothesis is incorrectly rejected. For a given test, the probability of obtaining a Type-I error is equal to the level of the test. When making multiple statistical tests, it therefore becomes increasingly probable that a Type-I error will be obtained. To preserve the rate of Type-I errors across multiple tests, a Bonferroni adjustment may be applied to the level of the test (or the corresponding critical value of the test statistic). The Bonferroni adjustment makes each of the multiple tests more stringent, such that the Type-I error rate for the family of tests will be set at the chosen test level [22]. In making *C* comparisons, this amounts to using an adjusted test level of *α*_*b*_=*α*/*C*. This Bonferroni adjustment is used to set the test levels in the hypothesis tests in §4c,d.

**(b) Standardized residuals of contingency tables**

A *χ*^2^-test of homogeneity may suggest that the observed data are inconsistent with a null hypothesis, but the test gives no information as to why the data are inconsistent. Agresti [20] describes how a cell-wise comparison of observed and expected frequencies in a contingency table can be used to better understand the results of a *χ*^2^-test. This is performed by computing the standardized residuals (*r*_*ij*_) between the observed occurrence (*n*_*ij*_) and expected occurrence under the null hypothesis of homogeneity (), using eqn 2.9 of [20],

A 1

where subscripts *i* and *j* refer to row and column indices, and *p*_*i*+_ and *p*_+*j*_ are the total proportion of the data in each row and column, respectively. Under the assumption of the null hypothesis, the standardized residuals are approximately normally distributed with zero mean and unit variance [20]. The standardized residuals are converted to *p*-values using the standard normal distribution, where they can be compared with a Bonferroni-adjusted *p*-value to identify cells that appear to be inconsistent with the null hypothesis of homogeneity [22]. For the 2×4 contingency table analysed in §4d there are eight comparisons, and so to maintain a Type-I error rate of 0.05, the adjusted *p*-value is 0.006.
